# Targeting RAB7 in human B lymphoma by a small molecule inhibitor arrests tumor cell growth

**DOI:** 10.3389/fonc.2025.1616519

**Published:** 2025-09-22

**Authors:** Maria Fernandez, Rui Wang, Jingwei Wang, Shuai Wu, Kenneth Holder, Alia Nazarullah, Ricardo C. T. Aguiar, Zijun Y. Xu-Monette, Ken H. Young, Hui Yan, Zhenming Xu

**Affiliations:** ^1^ Department of Microbiology, Immunology and Molecular Genetics, Joe R. & Teresa Lozano Long School of Medicine, The University of Texas at San Antonio, San Antonio, TX, United States; ^2^ Department of Pathology, Joe R. & Teresa Lozano Long School of Medicine, The University of Texas at San Antonio, San Antonio, TX, United States; ^3^ Department of Medicine, University of Texas Long School of Medicine, San Antonio, TX, United States; ^4^ South Texas Veterans Health Care System, Audie Murphy VA Hospital, San Antonio, TX, United States; ^5^ Hematopathology Division and Department of Pathology, Duke University Medical Center, Durham, NC, United States; ^6^ Duke Cancer Institute, Durham, NC, United States

**Keywords:** B cell lymphoma, Burkitt lymphoma, CID1067700, diffuse large B-cell lymphoma, FX1, MβCD, NF-κB, RAB7

## Abstract

RAB7, encoded by *RAB7A* in humans and *Rab7* in mice, is a small GTPase that catalyzes endosome maturation. It mediates NF-κB activation through the assembly of intracellular membrane signalosomes in stimulated normal B cells and plays a B cell-intrinsic role in the antibody response in mice. Here we show *RAB7A* transcripts are expressed in primary diffuse large B-cell lymphomas (DLBCLs), and that RAB7 protein expression is heightened in activated human tonsil B cells as well as in DLBCL and Burkitt lymphoma cell lines. Treating these cell lines with CID1067700, a selective small-molecule RAB7 inhibitor, results in a dose-dependent decrease in cell growth, associated with impaired proliferation and survival. CID1067700 also suppressed tumor development from Daudi cells, a Burkitt lymphoma cell line, in *Foxn1^nu/nu^* nude mice. The inhibitory effect of CID1067700 on Daudi cell growth *in vitro* is further enhanced by methyl-β-cyclodextrin, which disrupts plasma membrane lipid rafts, and by FX1, a BCL6 inhibitor. These findings, together with the unfavorable prognosis of DLBCL patients showing high *RAB7A* expression, suggest that targeting RAB7 is a promising therapeutic approach for mature B cell-derived lymphomas.

## Introduction

Many B cell malignancies have less than 60% 10-year survival rate under current therapies (1–4). New therapeutic targets are urgently needed, particularly those that are involved in NF-κB activation, a key factor in B cell lymphoma pathogenesis and therapy resistance (5, 6). RAB7 is a small GTPase encoded by *RAB7A* in humans and *Rab7* in mice. In addition to serving as the marker of mature endosomes and early lysosomes, RAB7 mediates the generation of mature endosomes from immature endosomes that are marked by the RAB5A small GTPase through a “GTPase switch” process (7). RAB7-tethered mature endosomes play a key role in multiple cellular processes (8), such as autophagy, protein degradation and cargo trafficking, including egress of SARS-CoV-2 (9). In mice, RAB7 is expressed in naïve B cells and further upregulated in stimulated B cells and plays an important role in NF-κB activation through the canonical pathway (10–12). Such RAB7-dependent NF-κB activation promotes the expression of activation-induced cytidine deaminase (AID) and, therefore, immunoglobulin heavy chain class switch DNA recombination (CSR) and the maturation of the antibody response cells (10). Mechanistically, RAB7 stabilizes the interaction between endocytosed immune receptors, such as CD40 after engagement, and signal transducer TRAF6 on mature endosomes, leading to heightened IKK activation and, therefore, NF-κB activation (12). This endosomal signaling pathway is in addition to the signaling emanating from the receptors localized on the plasma membrane. Enforced B cell-restricted RAB7 overexpression further enhances NF-κB activation and antibody responses (12), showing that RAB7 is an integral component of the B-cell NF-κB activation machinery and prompting us to address the relevance of RAB7 to human B cell lymphoma.

Identified by high-throughput screening as the only compound that affected the binding of RAB7 to GTP and GDP (13), CID1067700 binds RAB7 at the GTP-binding site with an affinity much higher than its binding to other small GTPases (14). As suggested by our modeling, such affinity difference likely results from an additional CID1067700-binding site located on the opposite side of the GTP-binding site in RAB7. In murine B cells stimulated *in vitro*, CID1067700 does not affect RAB7 expression, but inhibits its GTPase activity, NF-κB activation, AID induction and CSR. It specifically targets RAB7, as its inhibitory activity is lost in *Rab7* conditional knockout B cells. Conversely, CSR in CID1067700-treated B cells can be rescued by enforced expression of RAB7, but not RAB2A, a putative off-target of CID1067700 (11). Treatment of C57 mice with CID1067700 inhibits CSR and antibody responses *in vivo.* Likewise, treating lupus-prone mice, in which *Rab7* expression is upregulated in B cells, with CID1067700 reduces pathogenic autoantibody levels, alleviates disease symptoms and prolongs survival (11).

Here we hypothesized that RAB7 was upregulated in mature B cell-derived lymphoma cells and targeting RAB7 using CID1067700 could interfere with B cell lymphomagenesis. To test this hypothesis, we measured RAB7 expression in primary diffuse large B cell lymphomas (DLBCLs) and human B lymphoma cell lines. We further analyzed the correlation bettwen the DLBCL patient survival and their *RAB7A* expression. We also determined the efficacy of CID1067700 in inhibiting B cell lymphoma cell growth *in vitro*, alone or combined with two additional compounds. Finally, we treated a mouse model of human B cell lymphoma with CID1067700 to address the therapeutic potential of this RAB7 inhibitor.

## Materials and methods

### DLBCL patient survival analysis

The clinical, pathological and molecular features of 498 untreated diffuse large B-cell lymphoma (DLBCL) were previously described by us (15–17). Their gene expression data from microarray analyses are publicly available (GSE31312). Overall survival (OS) was calculated from the time of diagnosis to the last follow-up or death due to any cause. Progression-free survival (PFS) was calculated from the time of diagnosis to disease progression (relapse or death) or last follow-up.

### Primary DLBCL biopsies and tonsil samples

Seventeen de-identified frozen biopsies from patients with untreated DLBCL of the germinal center B cell (GCB-DLBCL) or activated B cell (ABC-DLBCL) origin were obtained from an archive maintained by the Department of Pathology at the University of Texas Health Science Center at San Antonio (UTHSCSA). The clinical, pathological and molecular features of these tumors were previously described (18) and are listed in **Table 1**. Three de-identified surgically removed tonsils were obtained from the University Health System hospital with the donors’ consent. All studies were approved by the UTHSCSA institutional review board.

**Table 1:** Clinical features of the primary DLBCLs used in this study.

Tumor ID	Diagnosis	Site	CD3^+^ T cell infiltration^*^
3466	GCB	Lymph node	3+
5204	GCB	Stomach	1+
5782	GCB	Lymph node	3+
6697	GCB	Lymph node	1+
7295	ABC	Lymph node	2+
7518	GCB	Lymph node	1+
8225	GCB	Spleen	NA
8394	GCB	Lymph node	NA
8468	ABC	Lymph node	NA
8566	GCB	Lymph node	NA
8746	ABC	Lymph node	NA
8750	GCB	Lymph node	NA
8779	GCB	Lymph node	NA
8781	GCB	Muscle	NA
8960	ABC	Bone	NA
9397	ABC	Lymph node	NA
9424	ABC	Bone	NA

^*^1+, rare, diffusely scattered (<5% T‐cells); 2+, small aggregates, focally abundant (5‐15% T‐cells); 3+, large aggregates, diffusely abundant (16‐25% T‐cells); NA, not available.

### Human naïve B cell enrichment and stimulation

Human naïve IgM^+^IgD^+^ B cells were purified by negative selection from peripheral blood mononuclear cells (PBMCs) obtained from healthy subject buffy coat (South Texas Blood & Tissue Center, San Antonio) using the EasySep Human Naïve B Cell Enrichment Kit (STEMCELL Technologies, cat# 19254). For activation, human naïve B cells (10^6^ cells/ml) were stimulated with CpG ODN2395 (2.5 mg/ml; invivogen), plus recombinant human IL-21 (50 ng/ml; BioLegend) for 48 hours, as described (19).

### Human tumor cell lines

B lymphoma cell lines included those established from Burkitt lymphoma (Daudi and CL-01), GCB-DLBCL (OCI-Ly7) or ABC-DLBCL (OCI-Ly10 and U2932). Other cell lines included Nalm-6 B leukemia, Jurkat T leukemia, SK-MEL-28 and UACC-62 melanoma, and U251 and U343 glioblastoma. All cell lines were cultured in RPMI 1640 medium supplemented with FBS (10% v/v, ThermoFisher) and penicillin-streptomycin/amphotericin B (1% v/v) (RPMI-FBS) at 37°C with 5% CO_2_.

### Tumor cell engraftment and growth *in vivo*

Daudi cell viability was checked (>95% live cells) before subcutaneous injection of 3×10^6^ cells into *Foxn1^nu/nu^* nude mice (JAX, Stock# 002019). Tumor sizes were measured every week. Length × width × width × ½ is used to calculate the size of the tumor. Mice with a tumor over 2500 mm^3^ in size were euthanized after one more measurement. Mice were euthanized by gradual displacement of room air with CO_2_ at approximately 30% of the chamber volume per minute until respiration ceased. Death was confirmed by cervical dislocation. The protocol was in accordance with the rules and regulations of the Institutional Animal Care and Use Committee (IACUC) of UTHSCSA.

### RAB7 expression and NF-κB activation

Cell pellets (5×10^6^ cells) were washed twice with cold PBS and lysed by sonication in PBS plus 1% Tween-20. After the protein concentration in cell lysates was determined, 20 μg lysates from each sample were analyzed by SDS-PAGE and Western blotting using anti-RAB7 antibody (Abcam; cat# ab137029) and anti-β-actin (Cell Signaling Technology, CST; cat# 4970).

For flow cytometry analysis, cell pellets (1×10^6^ cells) were washed once in cold Hank’s Buffered Salt Solution (HBSS) with 1% BSA and stained with fixable viability dye (FVS; BioLegend) and fluorophore-labeled mAbs to CD19 (BD biosciences; cat# 553785) and IgD (BD; cat# 562518). The cells were fixed and stained intracellularly with fluorophore-labeled mAb to RAB7 (Abcam; cat# ab198337), total RELA (CST; cat# 9609) and phosphorylated RELA (CST; cat# 4886), following the instructions of the BD intracellular staining kit (cat# 554714). Dead (FVS^+^) cells were excluded from the analysis.

### Drug treatment

For *in vivo* treatment, CID1067700 (Glixx Laboratories) dissolved in DMSO (stock concentration 40 mM, 16 mg/ml) was diluted to the final volume of 50 μl and injected intraperitoneally (i. p.) into *Foxn1^nu/nu^* mice daily from day 7 to day 12 at the dose of 16 mg/kg body weight. Mice injected i. p. with the vehicle DMSO (nil; 50 μl) showed no difference in tumor progression compared to those without any injection (data not shown). For *in vitro* treatment, the CID1067700 stock solution was diluted in DMSO to make 100× working solutions and then added to cell cultures to indicated final concentrations. To treat cells with methyl-β-cyclodextrin (MβCD, Sigma Aldrich), MβCD was freshly dissolved in RPMI-FBS to make a stock solution of 16 mM (21 mg/ml) and then further diluted with RPMI-FBS to make 4× working solutions before adding to cell cultures. To treat cells with FX1 (Selleck Chemicals), 10 mM solution in DMSO was diluted with RPMI-FBS to make 4× working solutions before adding to cell cultures.

### Real-time qRT-PCR transcript analysis

RNA was extracted from biopsies or B cells (2×10^6^) using the RNeasy Mini Kit (Qiagen). First-strand complementary DNA was synthesized from equal amounts of total RNA (4 μg) using the SuperScript III System (Invitrogen) and analyzed by the SYBR Green dye incorporation. The ΔΔCt method was used to determine transcript levels after normalizing to that of *ACTB* (encoding β-actin). qPCR primers used are:

*RAB7A*: forward, 5’-ACAGGCTAGTCACGATGCAG-3’, reverse, 5’-TTGGGGGCAGTCACATCAAA-3’;
*BCL6*: forward, 5’-GTTGTGGACACTTGCCGGAA-3’, reverse, 5’-CTCTTCACGAGGAGGCTTGAT-3’;
*PRDM1*: forward, 5’-GTGTCAGAACGGGATGAAC-3’, reverse, 5’-TGTTAGAACGGTAGAGGTCC-3’;
*CD79B*: forward, 5’-AGGGCCTGGACATTGACCA-3’, reverse, 5’-CACCTACAGACCACTTCACTTC-3’; ACTB: forward, 5’-AGAGCTACGAGCTGCCTGAC-3’, reverse, 5’-GCACTGTGTTGGCGTACAG-3’.

### MTS assay

Cells were seeded at 0.5×10^6^ cells/ml in RPMI-FBS and cultured for 24 hours and then re-seeded at 0.5×10^6^ cells/ml in 100 µl RPMI-FBS in the presence of drug or vehicle in a flat-bottom 96-well plate. After 24 or 48 hours, 20 µl MTS ([3-(4,5-dimethylthiazol-2-yl)-5-(3-carboxymethoxyphenyl)-2-(4-sulfophenyl)-2H-tetrazolium]; Promega; cat# G5421) was added. The plate was read 4 hours later at the 490 nm and 670 nm wavelengths. After subtracting the reading at 670 nm, the reading at 490 nm was used to measure cell growth after further subtraction of the background levels from RPMI-FBS medium only.

### Cell proliferation and viability analysis

B cells were labeled with carboxyfluorescein succinimidyl ester (CFSE) (ThermoFisher) following the manufacturer’s instructions and then cultured for 96 h. Cells were analyzed by flow cytometry for the CFSE intensity, which was reduced by half after completion of each cell division, when CFSE-labeled cell constituents were equally distributed between daughter cells. The FlowJo^®^ software was used to determine the number of B cell divisions that B cells had completed. The viability was analyzed by staining cells with 7-AAD followed by FACS to quantify 7-AAD^–^ live cells.

### Statistical analysis

DLBCL survival rate difference between two groups were compared using the Kaplan-Meier method and log-rank test in GraphPad Prism 10 software. Student *t* test or ANOVA was performed to compare group mean. The mixed linear effect model test was used to analyze the *in vivo* tumor growth and dose-dependent cell survival in combined treatments of CID1067700 with FX1 or MβCD.

## Results

### RAB7 expression in primary human B cells

**Figure 1:**
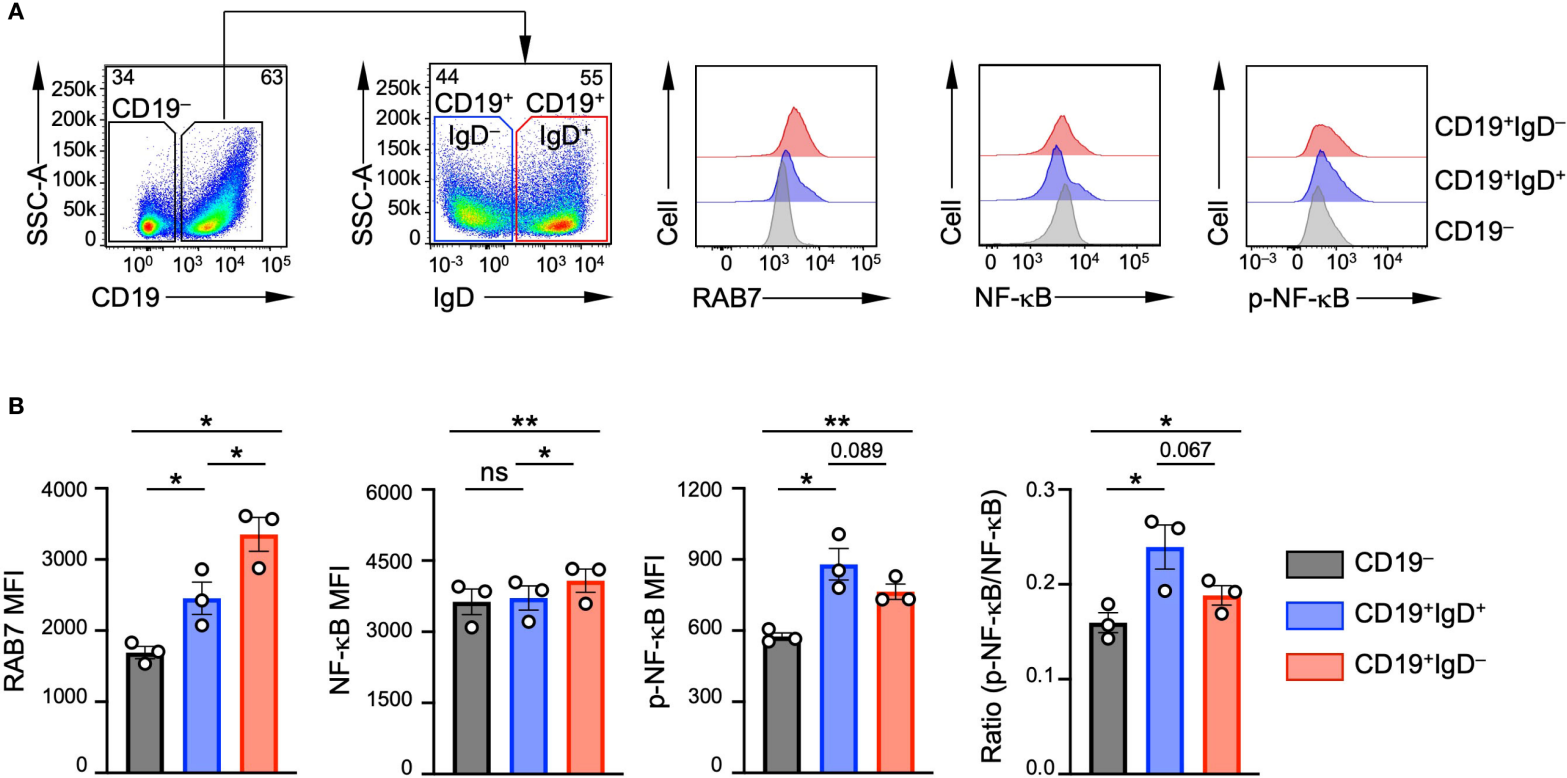
RAB7 expression in human tonsil B cells. **(A)** Representative flow cytometry plots showing the gating strategy used to identify CD19^-^ non-B cells, CD19^+^IgD^+^ and CD19^+^IgD^-^ B cell subsets from human tonsil samples (left) and the levels of RAB7, NF-κB RELA subunit and phosphorylated NF-κB RELA. **(B)** Quantification of the mean fluorescence intensity (MFI) of RAB7, NF-κB RELA, phosphorylated NF-κB RELA and the ratio of phosphorylated NF-κB over total NF-κB in different immune cell and B cell populations (n=3; mean and s.d.). *p < 0.05, **p < 0.01; one-way ANOVA.

As RAB7 is expressed by murine naïve B cells and further upregulated in activated B cells, we examined its expression in human B cells. RAB7 was expressed at higher levels in tonsil CD19^+^IgD^+^ B cells (primarily naïve with some activated IgD^+^ cells) and CD19^+^IgD^–^ B cells (activated/switching B cells and switched memory B cells) than in CD19^–^ non-B cells (**Figures 1A, B**). RAB7 expression was also higher in IgD^–^ B cells than in IgD^+^ B cells. Both B cell subsets displayed similar levels of the RELA (p65) subunit of NF-κB to CD19^–^ non-B cells, but higher levels of RELA phosphorylation at Ser536, an indicator of NF-κB activation, than non-B cells (**Figures 1A, B**). They, however, showed no significant difference in RELA phosphorylation or in the ratio of phosphorylate to total RELA (**Figures 1A, B**), likely reflecting the role of NF-κB activation in both B cell development and differentiation. Overall, RAB7 was expressed in primary human B lineage cells, including activated B cells.

### RAB7 expression in primary DLBCLs and human B lymphoma lines

**Figure 2:**
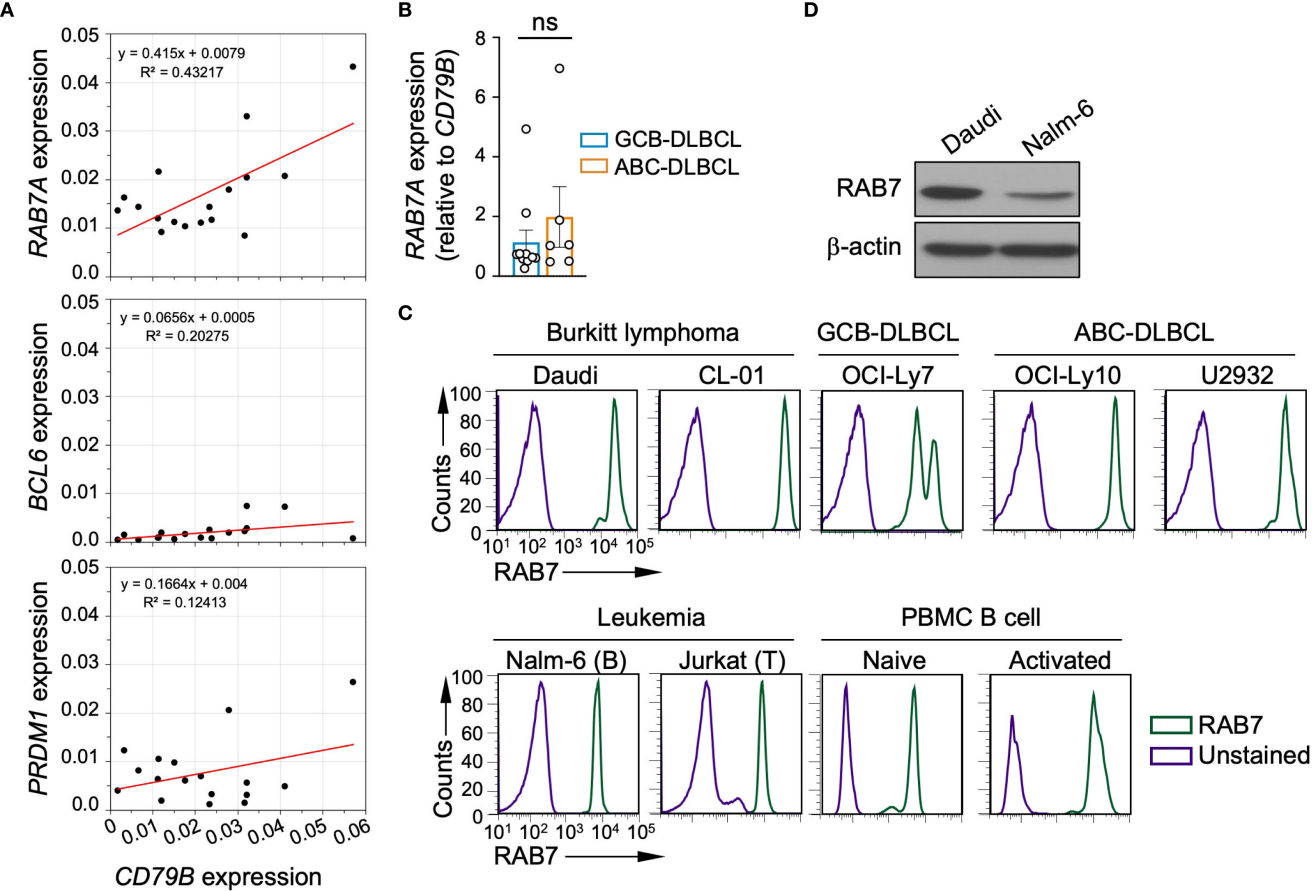
RAB7 transcript and protein expression in primary DLBCL and RAB7 in B-cell lymphoma. **(A)** Expression of *RAB7A, BCL6* and *PRDM1*, as measured by qRT-PCR and normalized to that of *ACTB* in 17 primary DLBCL and their relationship with the expression of *CD79B*. **(B)** *RAB7A* expression, as normalized to that of *CD79B* in 11 GCB-DLBCL and 6 ABC-DLBCL (mean and s.e.m.). ns, two-tailed unpaired *t-*test. **(C)** Flow cytometry analysis of levels of RAB7 in a panel of human B cell lymphoma and leukemia cell lines, as indicated, as well as naïve CD19^+^ B cells isolated from PBMCs before and after activation by CpG plus IL-21. Unstained cells were also analyzed as controls. Representative of 3 independent experiments. **(D)** Western blot analysis of RAB7 protein expression in Daudi and Nalm-6 cells. Representative of 3 independent experiments.

Prompted by heightened RAB7 expression is hyperactivated mature B cells, such as human and murine lupus B cells (11), we tested the hypothesis that RAB7 was also upregulated in mature B cell-derived lymphoma cells. We first analyzed *RAB7A* expression in biopsy samples from a small cohort of 17 DLBCL patients with well-defined clinical, hematological and pathological features (**Table 1**). The transcript level *RAB7A* was readily detectable in all samples, as were those of *BCL6*, a proto-oncogene frequently overexpressed in GCB-DLBCL, and *PRDM1*, a tumor suppressor gene frequently downregulated in ABC-DLBCL (**Figure 2A**). Notably, the expression of *RAB7A*, but not *BCL6* or *PRDM1*, correlated strongly with *CD79B*, a B-cell hallmark gene (**Figure 2A**), suggesting that malignant B cells, rather than tumor-infiltrating non-B cells, were the major contributors to *RAB7A* expression. In addition, *RAB7A* expression was similar in GCB-DLBCLs (n=11) and ABC-DLBCLs (n=6) at similar levels, indicating that RAB7 was broadly involved in most DLBCLs (**Figure 2B**).

To further confirm high RAB7 expression in malignant B cells, we analyzed a panel of cell lines derived from human mature B cell lymphomas, including Burkitt lymphoma, GCB-DLBCL and ABC-DLBCL. Among these, the Daudi and CL-01 Burkitt lymphoma cells as well as the OCI-Ly10 and U2932 ABC-DLBCL cells expressed RAB7 at high levels, whereas the OCI-Ly7 GCB-DLBCL cells exhibited a double-peak and relatively lower RAB7 expression pattern in flow cytometry analysis (**Figure 2C**). As comparisons, the Nalm-6 B leukemia and Jurkat T leukemia cell lines showed low RAB7 expression. The differential RAB7 expression in Daudi and Nalm-6 cells was confirmed by Western blotting (**Figure 2D**). Finally, RAB7 expression in B lymphoma cell lines was higher than that in B cells isolated from the PBMC of healthy donors and activated by TLR9 ligand CpG plus IL-21, and even higher than in naïve B cells (**Figure 2C**).

Thus, RAB7 was expressed in mature B cell-derived lymphomas irrespective the origin of the malignancies, at levels higher than those in normal B cells.

### *RAB7A* expression for DLBCL prognosis

**Figure 3:**
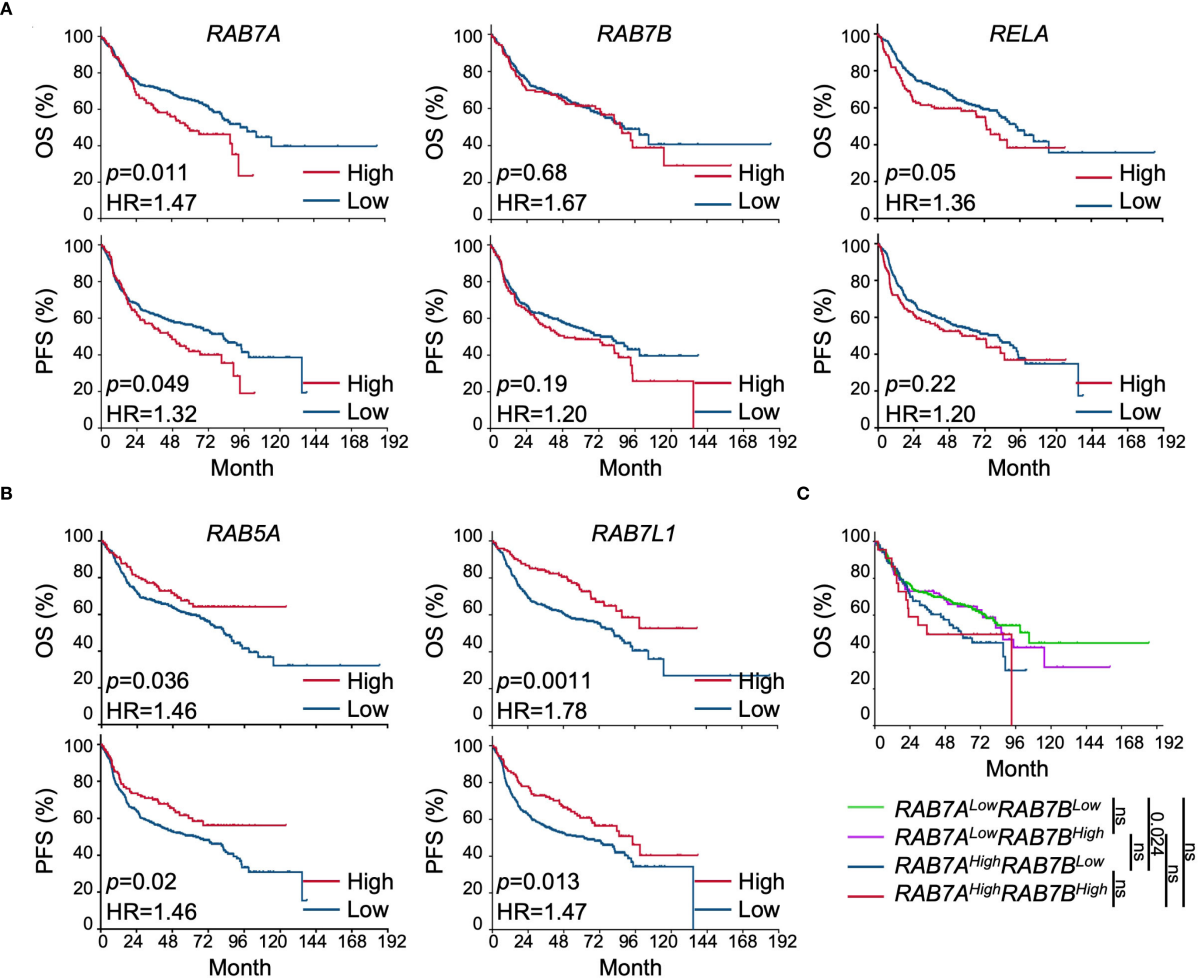
Correlation of *RAB7A* expression and DLBCL disease prognosis. **(A)** 15-year OS (top) and PFS (bottom) of DLBCL patients showing top-quartile expression (High) of *RAB7A, RAB7B* or *NFKB1* and the rest (75%) in the cohort (Low). **(B)** 15-year OS (top) and PFS (bottom) of DLBCL patients with high (top 25%) or low (bottom 75%) expression of *RAB5A* or *RAB7L1*. **(C)** 15-year OS of DLBCL patients displaying high (top 25%) or low (bottom 75%) expression of *RAB7A* and *RAB7B*. ns, *p>*0.5. In PFS, *p* values of the difference between any two groups were all over 0.05 (not shown).

Prompted by the heightened RAB7 expression in primary DLBCLs and B lymphoma cell lines, we next tested the hypothesis that higher RAB7 expression was associated with unfavorable outcomes in DLBCL. Using mRNA microarray data (GSE31312) of a mid-sized (n=498) cohort of DLBCLs, we analyzed the correlation between the expression of selected genes prior to treatment and patient survival. Within this cohort, the *RAB7A^High^* group exhibited significantly reduced OS (*p* = 0.011), with a reduction in PFS also approaching significant (*p* = 0.048) – PFS is an indicator of therapeutic resistance (**Figure 3A**). By comparison, high transcript levels of *RAB7B*, which encodes a homolog of RAB7 (50% homology), were not associated with reduced survival. The reduction in OS in the *RELA^High^* group compared with the *RELA^Low^* group approached significant (*p* = 0.05). In contrast to *RAB7A*, high expression of *RAB5A* was associated with better survival (**Figure 3B**), possibly reflecting heightened RAB5A activity that might inhibit the biogenesis of RAB7^+^ mature endosomes from RAB5A^+^ immature endosomes. High expression of *RAB7L1* was also associated with better survival, possibly reflecting increased diversion of RAB7^+^ mature endosomes into the endosome-Golgi pathway, leading to reduced RAB7 activity. We also divided the cohort into four groups, *RAB7A^High^RAB7B^High^*, *RAB7A^High^RAB7B^Low^*, *RAB7A^Low^RAB7B^High^* and *RAB7A^Low^RAB7B^Low^*. The *RAB7A^Low^RAB7B^Low^* group displayed better OS compared to the *RAB7A^High^RAB7B^Low^* group (**Figure 3C**). This, together with the lack of the significant difference between the *RAB7A^Low^RAB7B^High^* and *RAB7A^High^RAB7B^High^* groups, suggested that high *RAB7B* levels might narrow the difference in OS caused by differential *RAB7A* expression, probably due to compensatory effects.

In summary, *RAB7A* expression was associated with unfavorable prognosis in DLBCL, suggesting that RAB7 played a role in promoting DLBCL pathogenesis and/or therapeutic resistance.

### RAB7 inhibitor arrests B lymphoma cell growth *in vitro*

**Figure 4:**
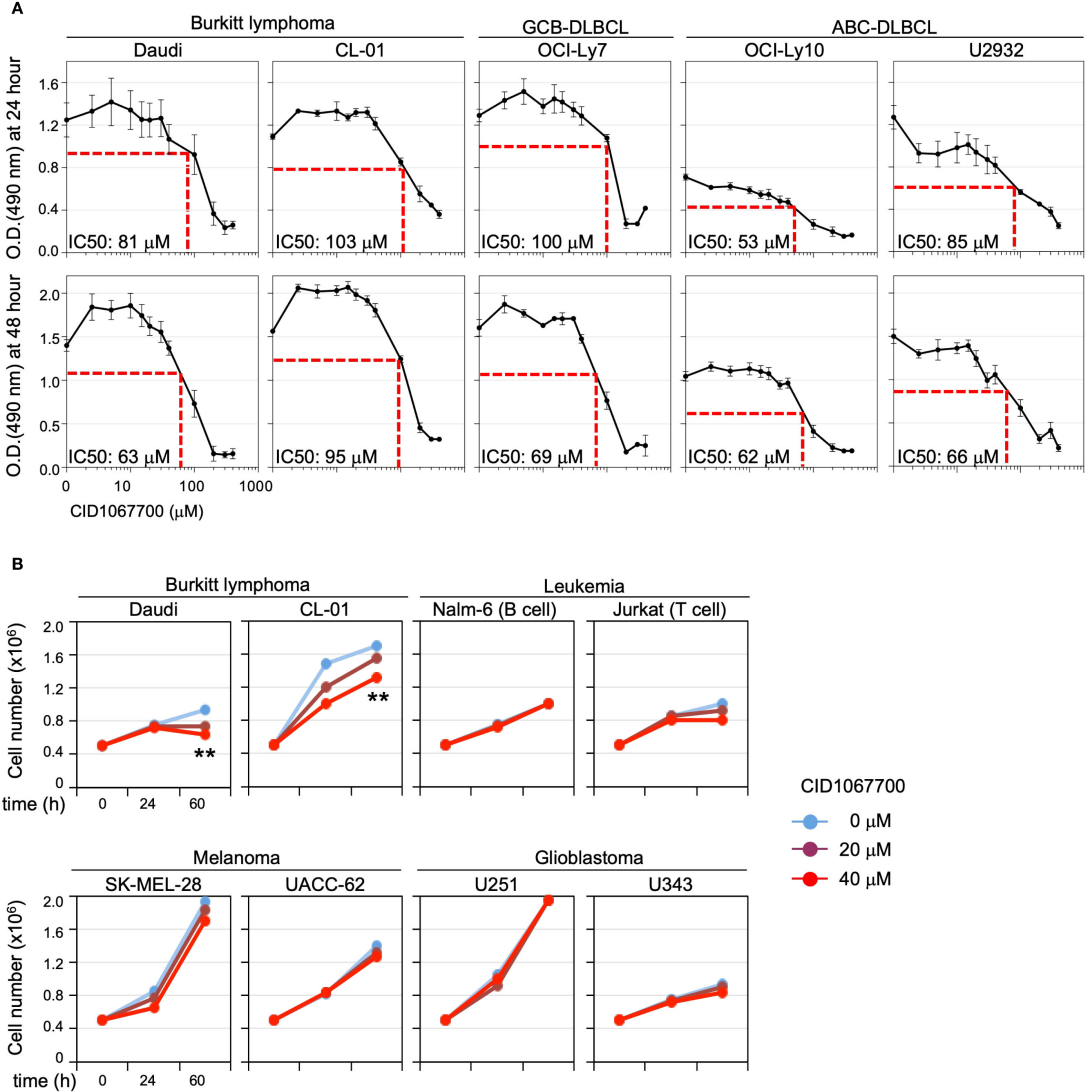
Effect of CID1067700 in arresting B cell lymphoma growth *in vitro*. **(A)** CID1067700 dose-dependent inhibition of the growth of B cell lymphoma cell lines, as indicated, after culturing for 24 (top) or 48 (bottom) hours, as analyzed by the MTS assay. IC50 values were calculated for each cell line based on the mean of replicates at each data points as follows: Daudi n=12; CL-01 n=3; OCI-Ly7 n=3; OCI-Ly10 n=12; U2932 n=3 (error bar, s.e.m. of three independent experiments). **(B)** Cell counts of B lymphoma cell lines as well as leukemia, melanoma and glioblastoma cell lines that were seeded at 0.5×10^6^ cells/ml and cultured for 24 and 60 hours in the absence of presence of 20 μM or 40 μM CID1067700 for 0, 24, and 60 hours. Each data point is the average of three technical replicates in one of the two independent experiments. **p < 0.01; two-way ANOVA.

To explore whether targeting RAB7, as highly expressed in B lymphoma cell lines, could have therapeutic effects, we first treated these cells *in vitro* with different doses of CID1067700 and assessed viability using the MTS assay at 24 and 48 hours. At 24 hours, all five cell lines, Daudi, CL-01, OCI-Ly7, OCI-Ly10 and U2932, exhibited a dose-dependent reduction in growth, with IC50 values ranging from 53 μM to 103 μM (**Figure 4A**). At 48 hours, the IC50 values slightly decreased in all cell lines except OCI-Ly10, ranging from 62 μM to 95 μM.

To determine whether the impact of CID1067700 was specific to B cell malignancies, we treated a panel of non-B lymphoma tumor cell lines, along with Daudi and CL-01 cells, with 0, 20 or 40 μM CID1067700 and monitored the total cell numbers at 24 hours and 60 hours. CID1067700 reduced the growth of Daudi and CL-01 cells in this assay, complementing the MTS results, which showed that 20 μM and 40 μM were the lowest effective doses of CID1067700 inhibiting the growth of these cells at 48 and 24 hours, respectively (**Figure 4B**). By contrast, Nalm-6 cells were resistant to the CID1067700 treatment and Jurkat cells showed a slight reduction only at 40 μM. Minimal to no inhibition was observed in SK-MEL-28 and UACC-62 melanoma cells or in U251 and U343 glioblastoma cells, indicating that the effects of CID1067700 were specific to B cell-derived lymphomas.

Taken together, the CID1067700 treatment arrested the *in vitro* growth of B cell lymphoma-derived cell lines, but not other cancer lines tested here.

### Inhibition of B cell lymphoma proliferation and survival by CID1067700

**Figure 5:**
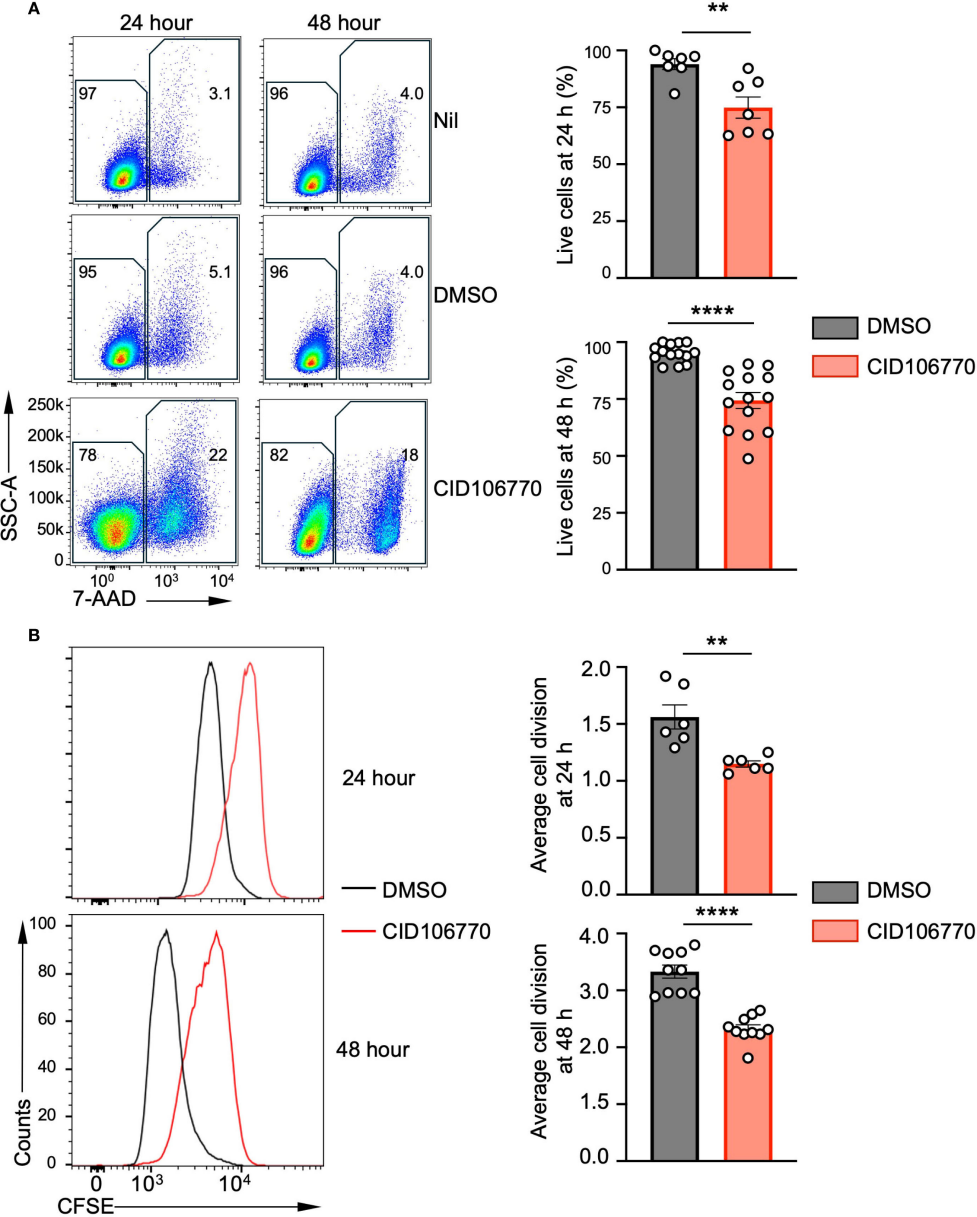
CID1067700 inhibits the survival and proliferation of Daudi cells. **(A)** Representative FACS plots of Daudi cells untreated (nil), treated with DMSO or CID1067700 (80 μM) for 24 (n=7) or 48 hours (n=14) after staining with 7-AAD to identify live cells (7-AAD^–^, left) and quantify them (right; mean and s.e.m.). **p < 0.01, ****p < 0.0001; two-tailed unpaired *t* test. **(B)** Representative FACS plots of Daudi cells labeled with CFSE, cultured for 24 (n=6) or 48 hours (n=10) in the presence of DMSO or CID1067700 (80 μM) and analyzed for CFSE intensities (left), as well as the quantification of average number of divisions based on halving of CFSE (right; mean and s.e.m.). FSC^low^SSC^low^ cells were inefficiently labeled by CFSE and, therefore, excluded from the depiction and the quantification of the average cell division. **p < 0.01, ****p < 0.0001; two-tailed unpaired *t* test.

To determine whether the reduced growth of B lymphoma cells observed with the CID1067700 treatment was due to increased cell death, impaired proliferation, or both, we treated Daudi cells with CID1067700 or vehicle (DMSO) for 24 and 48 hours. CID1067700 treatment resulted in a significant decrease in the proportion of live cells compared with DMSO treatment, to a comparable extent at both time points (**Figure 5A**), indicating that RAB7 inhibition reduced Daudi cell viability.

To investigate whether the RAB7 activity was required for B lymphoma cell proliferation, we analyzed Daudi cell divisions upon the CID1067700 treatment using the CFSE assay. At 24 hours, CID1067700 treatment resulted in a delay in CFSE halving, indicating impaired cell proliferation (**Figure 5B**, top). This was quantified by the decrease in the average cell division, from 1.5 in DMSO-treated control cells to 1.2 in CID1067700-treated cells, representing 20% reduction. At 48 hours, both CID1067700- and DMSO-treated Daudi cells exhibited further CFSE halving, with CID1067700-treated cells showing the average cell division of 2.2 compared to 3.3 in control cells (**Figure 5B**, bottom), a 33% reduction, more pronounced than that at 24 hours and likely contributing to the lower IC50 at 48 hours relative to 24 hours.

Overall, RAB7 inhibition in B lymphoma cells resulted in impaired viability and proliferation, both of which likely accounted for the arrested cell growth.

### Rab7 inhibition hampers tumor progression from engrafted Daudi cells *in vivo*

**Figure 6:**
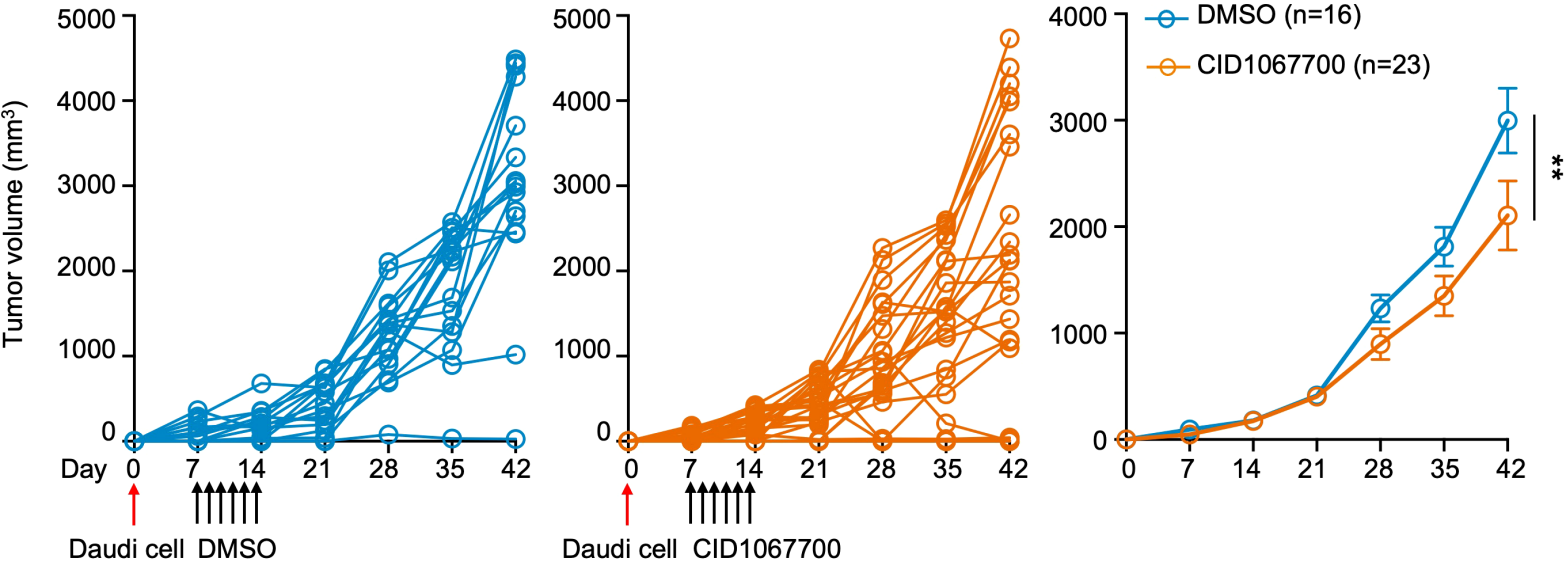
CID1067700 hampers Daudi B cell lymphoma progression *in vivo.* Spider graphs of tumor growth from Daudi cells (3×10^6^, >95% viability) engrafted subcutaneously in *Foxn1^nu/nu^* nude mice treated with DMSO (left) or CID1067700 (middle) six times, daily starting at day 8, through intraperitoneal injection. The mean and s.e.m. of the tumor volume from three independent experiments were depicted (right). **p < 0.01; mixed linear effect model.

We next assessed the effect of CID1067700 in a murine *in vivo* model of B cell lymphoma. Consistent with the low toxicity of CID1067700 previously reported in several mouse models by us (11) and others (20, 21), *Foxn1^nu/nu^* nude mice treated with CID1067700 at a dose of 16 mg/kg body weight did not show any overt signs of diseases (data not shown). After being engrafted with Daudi cells via the subcutaneous route, mice received 6 daily intraperitoneal injections of CID1067700 or DMSO starting on day 8. CID1067700-treated mice exhibited delayed tumor progression compared with control mice, with difference becoming apparent at day 28 (**Figure 6**), and no obvious side effects were observed during 42 days of monitoring (data not shown). These data showed that RAB7 inhibition arrested B lymphoma cell growth *in vivo* through a mechanism independent of T cell-mediated immunity.

### Enhancement of the suppressive effect of CID1067700 by MβCD and FX1

**Figure 7:**
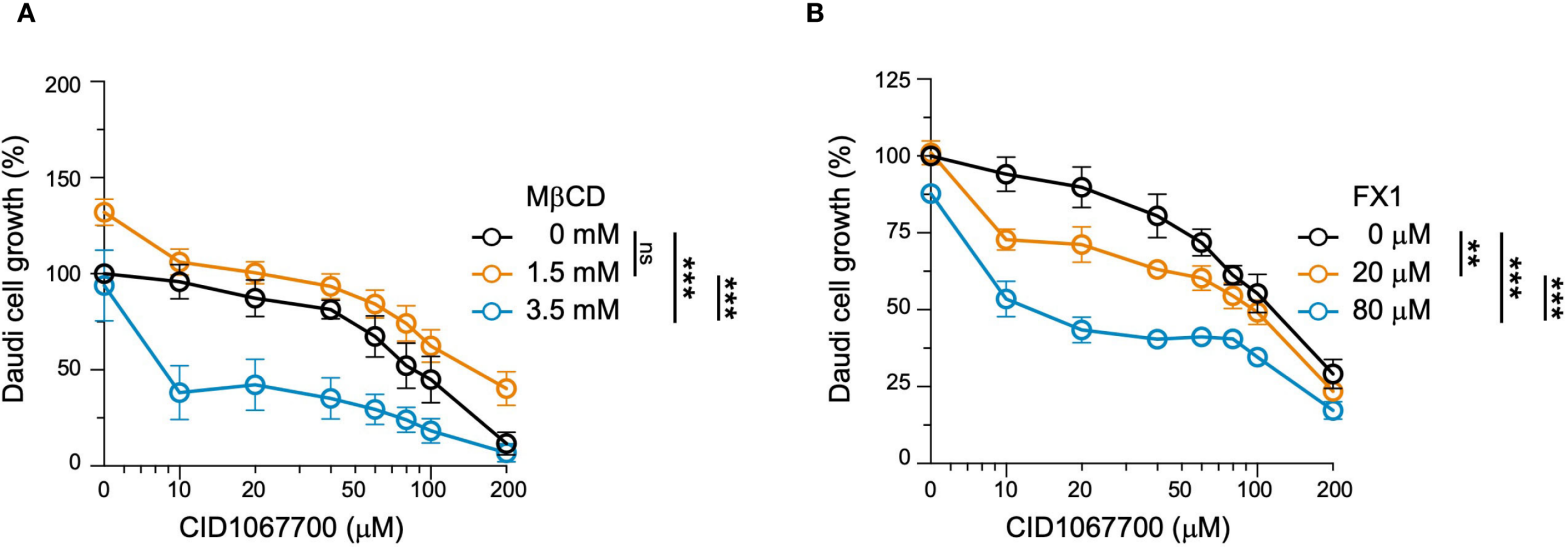
Enhancement of CID1067700-mediated inhibition of Daudi cell growth by MβCD and FX1. Daudi cell growth was assessed by the MTS assay after 24-hour co-treatment with varying concentrations of CID1067700, as indicated, plus vehicle or two different doses of **(A)** MβCD or **(B)** FX1, as indicated. The O.D.490nm values were normalized to those measured in cells without any drug treatment, set as 100% (n=3 in each data point; mean and s.e.m.). **p <0.01, ***p <0.001; mixed linear effect model.

We next investigated whether combination strategies could potentiate the suppression of Daudi cell growth by CID1067700. Our previous work has demonstrated that lipid rafts help anchor signaling receptors such as CD40 and TLR4 at the plasma membrane, where they initiate signal transduction. Treatment with MβCD, which disrupts these lipid rafts and associated CD40 signaling (22–24), diverts internalized receptors toward RAB7^+^ mature endosomes, where the multi-valent interactions among RAB7, CD40 and TRAF6 promote TRAF6 Lys63 polyubiquitination for heightened NF-κB activation in murine B cells (12). Accordingly, the combined treatment with MβCD and CID1067700 blocks both plasma membrane and intracellular membrane signaling pathways, leading to abrogated NF-κB activation and severely compromised B cell viability and differentiation (12). Treating Daudi B cells with 10 μM CID1067700 and 3.5 mM MβCD resulted in over 50% decrease in cell growth (**Figure 7A**). This indicated that the IC50 of CID1067700 was reduced to less than 10 μM in the presence of 3.5 mM MβCD, representing a more than eight-fold reduction compared with the IC50 of CID1067700 alone (80 μM). Combined treatment with a 1.5 mM MβCD, however, did not reduce the IC50. Notably, whereas treatment with 3.5 mM MβCD alone had no impact on Daudi cell growth, treatment with 1.5 mM MβCD alone increased the growth by 20%, similar to the increase in the NF-κB activation and CSR in murine B cells treated with MβCD alone, as previously shown (12).

FX1 is a selective BCL6 inhibitor identified through a rational design approach (25). Daudi cells treated with 80 μM or 20 μM FX1 displayed further decrease in the growth across nearly all CID1067700 doses tested (**Figure 7B**). This included 40% decrease when cells were treated with 80 μM FX1 and 10 μM CID1067700, resulting in an IC50 for CID1067700 slightly above 10 μM, which was still a significant reduction compared to the IC50 of CID1067700 alone. The FX1 treatment alone had only a marginal impact (**Figure 7B**).

In summary, inhibition of B cell lymphoma growth by the RAB7 inhibitor CID1067700 could be further enhanced by MβCD and FX1, which *per se* have no significant suppressive effects at the doses tested.

## Discussion

In this study, we identified a role for RAB7 in promoting B-cell lymphoma growth *in vitro*, in an animal model and potentially in primary DLBCL. Elevated RAB7 transcript and/or protein levels were observed in multiple lymphoma cell lines, including those from Burkitt lymphoma and two major DLBCL subtypes. The correlation between high *RAB7A* expression and poor OS in DLBCL patients, together with arrested B lymphoma cell growth *in vitro* and *in vivo* upon treatment by the RAB7 inhibitor CID1067700, would provide the rationale for the development of RAB7 inhibitors as new therapeutics.

The *RAB7A* expression pattern and the sensitivity to the CID1067700 treatment were not confined to a specific subtype of DLBCL, highlighting RAB7 as a promising drug target of ABC-DLBCL, which is more aggressive and more resistant to the current therapies than other forms of B cell lymphomas. This point is also relevant given the role of RAB7 in promoting NF-κB activation (10–12) and the central role of the constitutive NF-κB signaling in B cell lymphomagenesis, particularly ABC-DLBCL (26). Also consistent with a major role of NF-κB signaling in B cells in different pathophysiological contexts, B cells were the major contributors of *RAB7A* expression in lymphomas, as shown by the correlation between *RAB7A* and *CD79B* expression in primary DLBCLs and higher RAB7 protein levels in B lymphoma cell lines than in leukemia cell lines. Additional studies are needed to unveil the correlation between RAB7 expression and NF-κB activation in normal and malignant human primary B cells and to determine whether RAB7 promotes heightened NF-κB activation in human B cells through mechanisms similar to those observed in mouse B cells (12).

CID1067700 is a highly selective inhibitor of RAB7 and suppresses CSR in murine B cells by specifically targeting RAB7 (11). Whether it arrested human B lymphoma cell growth by exclusively targeting RAB7 remains to be investigated. This can be done by making cell lines with much reduced RAB7 expression using the CRISPR/Cas9 or siRNA/shRNA approaches and, conversely, by analyzing whether enforced expression of RAB7, but not putative off-target small GTPases, can rescue the lymphoma cell growth. Similar approaches can also be used to determine whether RAB7 inhibition by CID1067700 arrested cell growth primarily through NF-κB activation, e.g., by enforcing the expression of a constitutively active IKKβ mutant that induces canonical NF-κB hyperactivation to rescue lymphoma cell growth, similar to the rescue of CSR in CID1067700-treated mouse B cells by such an IKKβ mutant (11). In addition, *RAB7B*, which does not have a murine ortholog, might share a partial functional redundancy with *RAB7A* in human B cells. This notion would be consistent with the better OS of the *RAB7A^Low^RAB7B^Low^* group of DLBCL patients than the *RAB7A^High^RAB7B^Low^* group, but comparable OS between the *RAB7A^Low^RAB7B^High^* and *RAB7A^High^RAB7B^High^* groups. This potential redundancy might also contribute to the relatively high IC50 of CID1067700 in treating lymphoma cell lines *in vitro*, suggesting that dual RAB7/RAB7B inhibitors could be more potent therapeutics for B cell lymphomas.

Single-agent targeted therapies often fail due to pathway redundancy or compensatory effects. In addition to the possible compensation of general small GTPase activity of RAB7 mediated by RAB7B, the signaling-specific activity of RAB7 for NF-κB activation in B cells is redundant with signaling from the plasma membrane, where many immune receptors are anchored before being endocytosed upon engagement. Such redundancy might also contribute to the relatively high IC50 of CID1067700 alone (80 μM). Indeed, when abrogated by treatment with 3.5 mM MβCD, the IC50 was substantially reduced to less than 10 μM. As a transcription suppressor, BCL6 has a consensus DNA motif overlapping with that of NF-κB and, therefore, often antagonizes NF-κB in gene transcription (27). Thus, the BCL6 inhibitor FX1 reduced the IC50 of CID1067700 likely through a mechanism other than direct transcription regulation of target genes. In-depth mechanistic investigation might provide insights supporting the rationale to combine all three drugs to further decrease the IC50 of CID1067700.

As suggested by our data, malignant B cells were major contributors to *RAB7A* expression in primary DLBCLs, although *RAB7A* might also be expressed by normal B cells that could infiltrate DLBCL (28). Compared with the already upregulated RAB7 expression in stimulated primary human B cells, RAB7 protein levels in B lymphoma cells were even higher, suggesting a greater demand of these cancer cells for RAB7 and likely RAB7-dependent NF-κB activation. Such an “addiction” for RAB7 and NF-κB is also displayed by lupus B cells, as we have previously shown (11). The differential requirements for RAB7 by normal B cells and hyperactive malignant and lupus B cells also created a dose window within which RAB7 inhibition could arrest tumor growth or lupus disease development, without interfering with the function of normal B cells. Indeed, the 16 mg/kg body weight dose at which CID1067700 showed the inhibitory effect *in vivo* did not cause any overt disease symptoms and falls within the effective dose range of many clinically drugs. Further studies are needed to establish the toxicity profile of this compound and its future derivatives for potential use in combination therapies for relapsed B-cell lymphomas, where existing treatment options are limited.

## Data Availability

The original contributions presented in the study are included in the article/supplementary material. Further inquiries can be directed to the corresponding author.
