# Correction: Targeting RAB7 in human B lymphoma by a small molecule inhibitor arrests tumor cell growth

**DOI:** 10.3389/fonc.2025.1711784

**Published:** 2025-10-06

**Authors:** Maria Fernandez, Rui Wang, Jingwei Wang, Shuai Wu, Kenneth Holder, Alia Nazarullah, Ricardo C. T. Aguiar, Zijun Y. Xu-Monette, Ken H. Young, Hui Yan, Zhenming Xu

**Affiliations:** ^1^ Department of Microbiology, Immunology and Molecular Genetics, Joe R. & Teresa Lozano Long School of Medicine, The University of Texas at San Antonio, San Antonio, TX, United States; ^2^ Department of Pathology, Joe R. & Teresa Lozano Long School of Medicine, The University of Texas at San Antonio, San Antonio, TX, United States; ^3^ Department of Medicine, University of Texas Long School of Medicine, San Antonio, TX, United States; ^4^ South Texas Veterans Health Care System, Audie Murphy VA Hospital, San Antonio, TX, United States; ^5^ Hematopathology Division and Department of Pathology, Duke University Medical Center, Durham, NC, United States; ^6^ Duke Cancer Institute, Durham, NC, United States

**Keywords:** B cell lymphoma, Burkitt lymphoma, CID1067700, diffuse large B-cell lymphoma, FX1, MβCD, NF-κB, RAB7

The “NIH grants R01ES031522, R01GM140456, VA grants I01BX001882 and IK6BX006542 (to RCTA)” were erroneously omitted. The original version of this article has been updated.

